# Supplementary material for: Allosteric regulation and crystallographic fragment screening of SARS-CoV-2 NSP15 endoribonuclease
Source: Nucleic Acids Res. 2023 Apr 28;51(10):5255–70. doi: 10.1093/nar/gkad314 (PMC10250223; doi:10.1093/nar/gkad314)
Supplement: gkad314_Supplemental_Files [file gkad314_supplemental_files.zip › Supplementary datasets.zip!/231/Refine_8/01-BUSTER/Cycle-5/shell.01/R_Rfree.html]

# Rxpct factors in resolution bins at cycle 0


|  |  |  |  |  |  |  |  |  |
| --- | --- | --- | --- | --- | --- | --- | --- | --- |
|  | **<d> (Å)**  Rxpct\_Work | Rxpct\_Free | Rxpct\_All | |  |  |  |  |
|  | 13.1576 | 0.3388 | 0.3955 | 0.3412 |  |
|  | 7.5965 | 0.2830 | 0.3023 | 0.2842 |  |
|  | 5.8843 | 0.2684 | 0.2678 | 0.2684 |  |
|  | 4.9731 | 0.1975 | 0.2218 | 0.1989 |  |
|  | 4.3859 | 0.1799 | 0.1744 | 0.1796 |  |
|  | 3.9672 | 0.1864 | 0.2120 | 0.1876 |  |
|  | 3.6493 | 0.2051 | 0.2503 | 0.2073 |  |
|  | 3.3973 | 0.2051 | 0.2331 | 0.2066 |  |
|  | 3.1912 | 0.2048 | 0.2141 | 0.2052 |  |
|  | 3.0186 | 0.1956 | 0.2139 | 0.1964 |  |
|  | 2.8712 | 0.1960 | 0.2124 | 0.1968 |  |
|  | 2.7435 | 0.1871 | 0.2216 | 0.1888 |  |
|  | 2.6315 | 0.1898 | 0.2189 | 0.1916 |  |
|  | 2.5322 | 0.1851 | 0.1873 | 0.1852 |  |
|  | 2.4433 | 0.1848 | 0.1965 | 0.1853 |  |
|  | 2.3632 | 0.1908 | 0.1980 | 0.1912 |  |
|  | 2.2904 | 0.1889 | 0.1908 | 0.1890 |  |
|  | 2.2240 | 0.2044 | 0.2230 | 0.2053 |  |
|  | 2.1631 | 0.2066 | 0.2295 | 0.2077 |  |
|  | 2.1069 | 0.2475 | 0.2358 | 0.2468 |  |
|  | **Overall** | 0.2121 | 0.2303 | 0.2130 |  |

# Rxpct factors in resolution bins at cycle 1


|  |  |  |  |  |  |  |  |  |
| --- | --- | --- | --- | --- | --- | --- | --- | --- |
|  | **<d> (Å)**  Rxpct\_Work | Rxpct\_Free | Rxpct\_All | |  |  |  |  |
|  | 13.1576 | 0.3388 | 0.3955 | 0.3411 |  |
|  | 7.5965 | 0.2829 | 0.3022 | 0.2841 |  |
|  | 5.8843 | 0.2683 | 0.2678 | 0.2683 |  |
|  | 4.9731 | 0.1974 | 0.2217 | 0.1988 |  |
|  | 4.3859 | 0.1798 | 0.1744 | 0.1795 |  |
|  | 3.9672 | 0.1864 | 0.2119 | 0.1876 |  |
|  | 3.6493 | 0.2050 | 0.2502 | 0.2072 |  |
|  | 3.3973 | 0.2050 | 0.2330 | 0.2065 |  |
|  | 3.1912 | 0.2046 | 0.2140 | 0.2051 |  |
|  | 3.0186 | 0.1955 | 0.2138 | 0.1963 |  |
|  | 2.8712 | 0.1959 | 0.2125 | 0.1967 |  |
|  | 2.7435 | 0.1870 | 0.2215 | 0.1887 |  |
|  | 2.6315 | 0.1897 | 0.2189 | 0.1915 |  |
|  | 2.5322 | 0.1850 | 0.1872 | 0.1852 |  |
|  | 2.4433 | 0.1848 | 0.1965 | 0.1853 |  |
|  | 2.3632 | 0.1908 | 0.1981 | 0.1912 |  |
|  | 2.2904 | 0.1889 | 0.1907 | 0.1890 |  |
|  | 2.2240 | 0.2044 | 0.2229 | 0.2052 |  |
|  | 2.1631 | 0.2065 | 0.2294 | 0.2076 |  |
|  | 2.1069 | 0.2475 | 0.2359 | 0.2468 |  |
|  | **Overall** | 0.2120 | 0.2303 | 0.2129 |  |

# Rxpct factors in resolution bins at cycle 2


|  |  |  |  |  |  |  |  |  |
| --- | --- | --- | --- | --- | --- | --- | --- | --- |
|  | **<d> (Å)**  Rxpct\_Work | Rxpct\_Free | Rxpct\_All | |  |  |  |  |
|  | 13.1576 | 0.3386 | 0.3952 | 0.3409 |  |
|  | 7.5965 | 0.2826 | 0.3019 | 0.2837 |  |
|  | 5.8843 | 0.2678 | 0.2674 | 0.2678 |  |
|  | 4.9731 | 0.1971 | 0.2215 | 0.1985 |  |
|  | 4.3859 | 0.1796 | 0.1743 | 0.1793 |  |
|  | 3.9672 | 0.1861 | 0.2117 | 0.1874 |  |
|  | 3.6493 | 0.2047 | 0.2502 | 0.2070 |  |
|  | 3.3973 | 0.2046 | 0.2329 | 0.2062 |  |
|  | 3.1912 | 0.2043 | 0.2139 | 0.2048 |  |
|  | 3.0186 | 0.1953 | 0.2137 | 0.1961 |  |
|  | 2.8712 | 0.1957 | 0.2127 | 0.1965 |  |
|  | 2.7435 | 0.1868 | 0.2214 | 0.1885 |  |
|  | 2.6315 | 0.1896 | 0.2190 | 0.1914 |  |
|  | 2.5322 | 0.1850 | 0.1871 | 0.1851 |  |
|  | 2.4433 | 0.1848 | 0.1966 | 0.1854 |  |
|  | 2.3632 | 0.1908 | 0.1981 | 0.1912 |  |
|  | 2.2904 | 0.1889 | 0.1906 | 0.1890 |  |
|  | 2.2240 | 0.2044 | 0.2229 | 0.2052 |  |
|  | 2.1631 | 0.2065 | 0.2294 | 0.2076 |  |
|  | 2.1069 | 0.2475 | 0.2358 | 0.2468 |  |
|  | **Overall** | 0.2118 | 0.2302 | 0.2127 |  |

# Rxpct factors in resolution bins at cycle 3


|  |  |  |  |  |  |  |  |  |
| --- | --- | --- | --- | --- | --- | --- | --- | --- |
|  | **<d> (Å)**  Rxpct\_Work | Rxpct\_Free | Rxpct\_All | |  |  |  |  |
|  | 13.1576 | 0.3314 | 0.3948 | 0.3341 |  |
|  | 7.5965 | 0.2778 | 0.2966 | 0.2789 |  |
|  | 5.8843 | 0.2602 | 0.2642 | 0.2603 |  |
|  | 4.9731 | 0.1943 | 0.2137 | 0.1954 |  |
|  | 4.3859 | 0.1774 | 0.1737 | 0.1772 |  |
|  | 3.9672 | 0.1845 | 0.2160 | 0.1860 |  |
|  | 3.6493 | 0.2024 | 0.2506 | 0.2048 |  |
|  | 3.3973 | 0.2014 | 0.2305 | 0.2029 |  |
|  | 3.1912 | 0.2022 | 0.2146 | 0.2029 |  |
|  | 3.0186 | 0.1937 | 0.2118 | 0.1945 |  |
|  | 2.8712 | 0.1939 | 0.2135 | 0.1948 |  |
|  | 2.7435 | 0.1862 | 0.2198 | 0.1879 |  |
|  | 2.6315 | 0.1891 | 0.2177 | 0.1908 |  |
|  | 2.5322 | 0.1854 | 0.1887 | 0.1856 |  |
|  | 2.4433 | 0.1851 | 0.1969 | 0.1857 |  |
|  | 2.3632 | 0.1913 | 0.1994 | 0.1917 |  |
|  | 2.2904 | 0.1894 | 0.1925 | 0.1895 |  |
|  | 2.2240 | 0.2050 | 0.2256 | 0.2059 |  |
|  | 2.1631 | 0.2068 | 0.2312 | 0.2080 |  |
|  | 2.1069 | 0.2484 | 0.2381 | 0.2478 |  |
|  | **Overall** | 0.2093 | 0.2291 | 0.2103 |  |
